# Report of Five Cases of Diphtheria, with Remarks

**Published:** 1889-03

**Authors:** F. A. Schmitt

**Affiliations:** Shulenburg, Texas


### REPORT OF FIVE CASES OF DIPHTHERIA, WITH REMARKS.

By F. A. Schmitt, M. D., Shulenburg, Texas.

[For Daniel's Texas Medical Journal.]

In a former paper—a translation from the German—the view was taken, that diphtheria is a local disease, becoming general afterwards ; that the diphtheritic poison finds a favorable "nidus" in the mucosa of the throat—there rapidly multiplying—and that constitutional symptoms supervene only after the epithelial barrier is broken down, the denuded surface allowing the poison free access into the circulation.

Five cases, all occurring in the same family—an epidemic on a small scale, as it were—two resulting fatally, three recovering, will give additional evidence as to this, and besides will demonstrate anew the necessity of early treatment.

The child of Mr. E., a farmer living six miles in the country, complained of sore throat. During the first 24 hours some household remedies were used. The child getting worse, it was decided to send for me, their family physician. Unfortunately was the messenger on entering town informed that I was absent from town and no other physician present. Instead of inquiring at my office, he proceeded to the nearest drug store to get a throat wash for the little patient. Thus many hours more were consumed, and when I arrived on the third or fourth day, the child was in a hopeless asthenic condition and died the next day.

The people were poor as far as wordly means are concerned, but they were recompensed by having quite a number of children, they ranging from the tender age of a few months to nine or ten years and all occupying and sleeping in the same room.

Although no others were complaining at the time, I took the precaution of examining their throats; failing to detect any abnormal condition in any of them, I contented myself by prescribing what may be termed a prophylatic remedy: R. Tr. Iron mur. Potass. chlor. aa two drachms, water four ounces; with directions to use as a gargle two or three times a day excepting only the infant.

Two weeks afterwards the second child was attacked, but owing to a creek being up, I could not be notified until late on the third day; even then it was hazardous for me to cross the stream, but I managed to get to the other side.

I found the little patient— a girl five years of age—in a worse condition than the first—dying, and two others feverish, with characteristic diphtheritic patches on their tonsils. I at once subjected those to rigid local and general treatment. A mixture, containing equal parts of Tr. Chlor. Iron, Glycerine, Chlor. Potass. and water I immediately applied with a sponge probang. Besides I gave them internally 10 drops of the same mixture but slightly diluted and to be repeated every two hours. Twenty-four hours afterwards fever and exudation had disappeared and no unfavorable symptoms supervened.

A week later I was again called to the bedside of another victim, but fortunately soon enough to leave a chance for a favorable termination. Although constitutional symptoms—the thermometer registered 102½—had supervened, and poisonous exudations visible over both tonsils, I had the pleasure of seeing the child recover.

I ordered the child to be given, and to be repeated every two hours, ten drops of Mur. Tr. of Iron, and six grains of quinine, in addition to topical treatment as above stated. On my next visit, sixteen hours afterwards, I found the child with marked cinchonism, but without fever and diphtheritic exudations, and in a fair way of recovery.

As there had been no similar case in the neighborhood, I could not trace the origin of the epidemic, but the cabin had formerly been occupied by negroes, some of whom, as my black informant told me, had died of throat disease. The cabin was undergoing repairs at the time, and the dormant germ may have been disturbed.

I conclude my report with a few remarks in regard to the "modus operandi" of the remedies used:

We know that nature attempts to interpose a barrier, by the reactive inflammation set up at the seat of the lodgement of the virus, by thickening the epithelium and by enlarging the nearest lymphatic glands so as to check filtration through their interior tubes ; but we also know that these efforts on the part of nature are often fertile or that these natural barriers in the course of time become broken down and the poison is given free access into the general circulation.

Reasoning thus, our efforts should be directed in endeavoring to assist nature in its wise, but futile attempts at interposition; and in my humble opinion is the action of the Tr. of Iron largely due to its coagulating influence, or in the language of Trousseau: "To the modification it exercises on the character of the surfaces covered with diphtheritic exudation." Again, we know, that quinia exerts the most powerful action of all the parasiticides in malaria, and we know by experience that large doses of this drug exercise a deleterious effect upon the poison of diphtheria.

If then, small quantities of the virus have gained access into the blood—ushered in by but slight acceleration of temperature,—we have by the administration of heroic—(comparatively) doses of quinine a probable means of ridding the blood of these few advanced invaders, and gain time to guard against renewed invasion.

